# Genetic Analysis of the Single-Nucleotide Polymorphisms rs880810, rs545793, rs80094639, and rs13251901 in Nonsyndromic Oral Clefts: A Case–Parent Trio Study

**DOI:** 10.1055/s-0043-1764399

**Published:** 2023-03-28

**Authors:** Mahamad Irfanulla Khan, Prashanth CS, N. Srinath, Praveen K. Neela, Mohammed K. Mohiuddin

**Affiliations:** 1 Department of Orthodontics and Dentofacial Orthopedics, The Oxford Dental College, Bangalore, Karnataka, India; 2 Department of Orthodontics and Dentofacial Orthopedics, D.A Pandu Memorial R.V Dental College, Bangalore, Karnataka, India; 3 Department of Oral and Maxillofacial Surgery, Krishnadevaraya College of Dental Sciences, Bangalore, Karnataka, India; 4 Department of Orthodontics and Dentofacial Orthopedics, Kamineni Institute of Dental Sciences, Narketpally, Telangana, India; 5 Multi-Disciplinary Research Unit, Osmania Medical College, Hyderabad, Telangana, India

**Keywords:** oral clefts, nonsyndromic, *PAX7*, gene, genotyping

## Abstract

Oral clefts, including cleft lip (CL), cleft palate (CP), and cleft lip and palate (CLP), are the most common types of congenital anomalies of the human face. Various genetic and environmental factors play a role in developing oral clefts. Several studies have shown the association of the *PAX7* gene and the 8q24 region with these oral clefts in different populations worldwide. However, there are no reported studies on the possible connection between the *PAX7* gene and the 8q24 region nucleotide variants and the risk of developing nonsyndromic oral clefts (NSOC) in the Indian population. Hence, this study aimed to test the possible association between *PAX7* gene single-nucleotide polymorphisms (SNPs) rs880810, rs545793,rs80094639, and rs13251901 of the 8q24 region using a case-parent trio design. Forty case-parent trios were selected from the CLP center. Genomic DNA was isolated from the cases and their parents. The rs880810, rs545793, rs80094639, and rs13251901 were genotyped by the MassARRAY technique. PLINK software was used for statistical analysis. All the SNPs were tested for Hardy-Weinberg equilibrium. No statistical significance was found with any SNPs, as none of the genotyped SNPs showed a *p* -value of less than 0.05. Hence, the rs880810, rs545793, and rs80094639 of the *PAX7* gene, and rs13251901 of the 8q24 region are not associated with NSOC in the Indian population.

## Introduction

Oral clefts, including cleft lip (CL), cleft palate (CP), and cleft lip and palate (CLP), are the most common congenital maxillofacial deformities 1 and form a major public health burden both in terms of medical expenses and disturbing to the patients and their families. 2 These oral clefts not only result in maxillofacial deformities but also occur with complications, such as regurgitation, middle ear infections, speech disorders, and other psychosocial problems. 3 4

The expression of oral clefts is usually unique and their occurrence varies by ethnicity, geography, and socioeconomic status. 5 Asians show higher prevalence rates, Caucasians show intermediate, and Africans show lower rates. 6 Literature suggests that 70% of oral cleft cases are nonsyndromic, whereas 30% are syndromic. 7 8

The paired box 7 ( *PAX7* ) gene has been indicated as a candidate gene for oral clefts. It also plays a role in neural crest development and maxillary growth. In addition, during craniofacial development, the *PAX7* involves in the regulation of morphogenesis, survival, patterning, and specification of the frontonasal structures. So, any embryological disturbance in the neural crest development may lead to the development of oral clefts such as cleft lip and cleft palate. 9 A small number of human studies are available in the literature on this gene. 10 11 12 Traditionally, case–control studies have been used to study the etiology of oral clefts. Alternate to the traditional case–control study is the case-parent trio design in which cleft patient and their parents are analyzed for the genotypes. 13

High-risk single-nucleotide polymorphisms (SNPs rs880810, rs545793, rs80094639 of the *PAX7* gene, and rs13251901 of the 8q24 region are involved in the etiology of nonsyndromic oral clefts (NSOC) have been evaluated in different populations worldwide. A literature review showed the SNP rs13251901 belongs 8q24 region to have gene–gene (GxG)interaction with *the PAX7* gene in cleft case-parent trios. Furthermore, several genomic studies have successfully replicated the associations between *PAX7* and NSOC in other populations like Singaporean, Korean, Taiwanese, Philippines, Japanese, and Chinese. 10 11 12 However, the current literature search shows no studies on the association between these SNPs with NSOC in the Indian population. Hence, in this study, we tested for the possible association between *PAX7* gene SNPsrs880810, rs545793, rs80094639, and rs13251901 of the 8q24 region in NSOC using a case-parent trio design. The advantages of the case-parent trio design include robustness in sample collection, testing maternal versus paternal effects, studying the parent-of-origin effects, and obtaining the accuracy of the genotyping.

## Materials and Methods

### Study Samples and Ethics Statement

Forty case-parent trios were selected from the CLP clinic of the Mallige Medical Centre, Bangalore. The sample size was estimated with a desired statistical power of 80% and a significance level of 0.05. Therefore, it was determined to include 40 case-parent trios. Geneticists examined each case to exclude the syndromic forms of oral clefts. The present research was approved by the Institutional Review Board (IRB) of DAPM RV Dental College, Bangalore (IRB no. 230/Vol-2/2017), and the research complied with the Helsinki declaration on medical research and ethics.

### Inclusion and Exclusion Criteria

Oral clefts, either having CL or CP, or CLP only, were included in this study. Geneticists screened cleft children for any syndromes or abnormalities. Patients with a congenital anomaly and any syndromes associated with cleft were excluded from the study. The mean age and standard deviation of the included study subjects are cleft patients (6.62 ± 5.30 years), father (36.07 ± 6.42 years), and mother (30.37 ± 5.45 years). Written informed consent was obtained from all the cases and their parents.

### SNP Selection, DNA Extraction, and Genotyping

The SNP srs880810, rs545793, rs80094639, and rs13251901 were selected from the previous genome-wide association studies, NCBI database( *www.ncbi.nlh.nih.gov/snp/* ) and Ensembl genome database ( *http://asia.ensembl.org/Homo_sapiens/Info/Index* ). Genomic DNA was extracted from 3 mL of peripheral blood using QIAamp DNA Mini Kit (Qiagen Inc, California, United States) following the manufactures instructions. All the SNPs were genotyped using the Agena Bio MassARRAY (Agena Bioscience, Inc., San Diego, California, United States) platform using matrix-assisted laser desorption/ionization-time of flight mass spectrometry. The genomic data acquired from the genotyping were evaluated using statistical tests.

### Statistical Data Analyses

All the case-parent trios underwent Hardy-Weinberg equilibrium (HWE) test, minor allele frequency (MAF) analysis, and the standard transmission disequilibrium test (TDT). The HWE, MAF, allelic TDT, and parent-of-origin effects in all the case-parent trios were calculated using PLINK software. 14 The 95% confidence interval for the assessed odds ratios was calculated, and a *p* -value less than 0.05 was set as statistically significant.

## Results

Forty trios were selected for this study, and Table 1 presents the demographic characteristics of study participants. The SNPs rs880810, rs545793, rs80094639, and rs13251901 ( Table 2 ) followed the HWE test. The family-based TDT results ( Table 3 ) showed no significant association with NSOC in the allelic frequencies of the Indian case-parent trios, as none of the genotyped SNPs showed a *p* -value less than 0.05.

**Table 1:** Summary of demographic variables

Sl. no.	Variables		Sample distribution *n* = 40 (%)
1	Age in years (mean ± standard deviation [SD])	Cleft patient	6.62 ± 5.30
		Father	36.07 ± 6.42
		Mother	30.37 ± 5.45
2	Gender of cleft patient	Male	15 (28%)
		Female	25 (72%)
3	Socioeconomic status	Lower middle class (III)	31 (72%)
		Lower class (V)	9 (28%)
4	Oral clefts	Cleft lip (CL)	13 (22%)
		Cleft palate (CP)	6 (16%)
		Cleft lip and palate (CLP)	21 (62%)

**Table 2:** List of the SNPs examined

Gene	SNP	Position	Call rate (%)	Alleles	Ancestral allele	Global MAF
*PAX7*	rs880810	1:18668060	100	C/G	C	0.11
*PAX7*	rs545793	1:18690028	100	G/A/C/T	G	0.34
*PAX7*	rs80094639	1:18723759	100	A/G	A	0.31
*8q24*	rs13251901	8:128287015	100	A/G	A	0.41

Abbreviations: A, adenine; G, guanine; C, cytosine; MAF, minor allele frequency (cleft cases); *PAX7* , paired box protein 7; SNP, single nucleotide polymorphism; T, thymine.

**Table 3:** Association between the *PAX7* gene SNPs with NSOC

Gene	SNP	A1	A2	OR	CHISQ	*p* -Value
*PAX7*	rs880810	G	C	3	1	0.31
*PAX7*	rs545793	G	C	1.5	3	0.08
*PAX7*	rs80094639	G	A	2	4	0.24
*8q24*	rs13251901	G	A	0.68	0.92	0.33

Abbreviations: A, adenine; A1, major allele (wild allele); A2, minor allele (variant); C, cytosine; CHISQ, chi-squared test; G, guanine; NSOC, nonsyndromic oral clefts; OR, odds ratio; *PAX7* , paired box protein 7; SNP, single nucleotide polymorphism; *p* -Value < 0.05 is significant.

## Discussion

Several genes and environmental factors play a significant role in the etiology of oral clefts. Advances in genetics and molecular biology techniques have discovered the genetic basis of these craniofacial defects. Numerous genes associated with the etiology of oral clefts have been discovered and registered in the Online Mendelian Inheritance in Man database. Different genetic approaches have been used to identify these genes causing oral clefts, including animal models, 15 linkage studies, 16 candidate gene analyses, 17 and genome-wide association studies. 18

Case-parent trio design studies are commonly used in genetics as an alternative to case–control studies. In these studies, the affected cleft children and their parents (father and mother) are genotyped. 19 Therefore, it does not require control samples because the untransmitted parental genotypes are used as “controls” for the transmitted alleles or genotypes. 20 In addition, the case–parent trio design has the advantage of testing maternal versus paternal effects. It separates these from the effects of the fetal genotype versus parental origin robustly. 21

The *PAX7* gene encodes specific DNA-binding transcription factors and is involved in neural crest development which is closely associated with cranial and maxillofacial development. In turn, any defect in neural crest development may cause the development of oral clefts. 22 The *PAX7* gene, along with *PAX3* , plays an essential role during craniofacial development in the regulation of morphogenesis, survival, patterning, and specification of the frontonasal structures. *PAX3/PAX7* double mutant mice showed impaired function against environment-related teratogenesis and exhibited more severe facial morphogenesis defects. The transcription factors of this PAX7 and *PAX3* help to maintain the proliferative cells during the development of embryonic and fetal muscles of the trunk and limbs.

Several studies have documented their findings regarding the 1p36.13 chromosomal region and shown the association of the *PAX7* gene in the development of oral clefts in different populations worldwide. Furthermore, considering the G × G interaction, the polymorphisms of the *PAX7* showed a significant association with NSOC in some populations. But most of the studies were largely focused on the Western population, 10 11 and there are no reported case-parent trio studies of the *PAX7* gene in the Indian population.

Hence, the SNP srs880810, rs545793, rs80094639, and rs13251901 of the 8q24 region were analyzed in the NSOC patients in a case-parent trio design; there is no reported trio study on the role of these SNPs in the Indian population. Forty case-parent trios were selected from the cleft center with cleft samples consisting of males (15) and females (25). The gender difference in our study samples was more compared to the global prevalence of NSOC in males and females. This study results suggest that rs880810, rs545793, rs80094639, and rs13251901 are not signiﬁcantly associated with NSOC.

In a study by Sull et al, 10 a total of 297 CLP trios from four different populations (Singaporean, Korean, Taiwanese, and Maryland) showed that the rs880810 and rs545793 of *PAX7* were not associated with NSOC.

Also, in multiplex families of CLP, the rs80094639 showed no significant association. 23 A study showed the SNP rs13251901 belongs 8q24 region to have GxG interaction with the *PAX7* gene in cleft case-parent trios and showed no significance with NSOC. 24

The difference or varying results related to these SNPs in the genetic etiology of oral cleft might be due to the different ethnicity, epigenetic factors, and environmental factors.

### Limitations

The limitations of our study are a relatively smaller sample size and the analysis of only four polymorphisms. Further studies should be conducted with more SNPs of the *PAX7* gene and the 8q24 region with a bigger sample size for the Indian population to substantiate their role in the etiology of NSOC.

## Conclusion

This study revealed that the SNPs rs880810, rs545793, rs80094639 of the PAX7 gene and rs13251901 of the 8q24 region are not associated with the risk of NSOC in the Indian population.
